# Suppression of ANT2 by miR-137 Inhibits Prostate Tumorigenesis

**DOI:** 10.3389/fgene.2021.687236

**Published:** 2021-09-03

**Authors:** Heyuan Zhang, Nanhui Chen, Zhihai Deng, Yang Mai, Limin Deng, Guo Chen, Yutong Li, Bin Pan, Weifeng Zhong

**Affiliations:** ^1^Department of Urology, Meizhou People’s Hospital (Huangtang Hospital), Meizhou, China; ^2^Guangdong Provincial Key Laboratory of Precision Medicine and Clinical Translational Research of Hakka Population, Meizhou People’s Hospital (Huangtang Hospital), Meizhou, China; ^3^Department of Urology, The First Affiliated Hospital of Jinan University, Guangzhou, China; ^4^Department of Urology, Guangzhou Twelfth People’s Hospital, Guangzhou, China; ^5^Department of Urology, Gaozhou People’s Hospital, Gaozhou, China

**Keywords:** miR-137, prostate cancer, DU145, LNCaP, growth, migration, invasion, ANT2

## Abstract

Prostate cancer (PCa) is a serious disease that affects men’s health. To date, no effective and long-lasting treatment option for this condition is available in clinical practice. ANT2 is highly expressed in a variety of hormone-related cancers, but its relationship and regulatory mechanism with PCa are unclear. In this study, we found that ANT2 expression was significantly upregulated in PCa tissues relative to control samples. Genetic knockdown of ANT2 effectively inhibited, while overexpression promoted, proliferation, migration, and invasion of PCa cells. In addition, miR-137 expression was reduced in prostate cancer tissues relative to control tissues. We identified a regulatory site for miR-137 in the 3′-UTR of ANT2 mRNA; luciferase reporter assays indicated that ANT2 is a direct target gene for miR-137. Transfecting cells with miR-137 mimics and/or an ANT2-encoding plasmid revealed that ANT2 promotes proliferation, migration, and invasion of PCa, whereas co-expression of miR-137 mimics inhibited these behaviors. These observations suggest that miR-137 mimics inhibit development of PCa by antagonizing expression of ANT2. Furthermore, tumorigenic assays in nude mice showed that miR-137 inhibitors abolished the inhibitory effect of ANT2 knockdown on PCa tumor growth. Collectively, our findings suggest that ANT2, a target gene of miR-137, is intimately involved in development of PCa, providing new evidence for the mechanism underlying pathogenesis of PCa as well as new options for targeted therapy.

## Introduction

Prostate cancer (PCa) is a very common cancer in men (Bray et al., 2018), almost 20% of whom have metastatic disease at the time of diagnosis (Wu et al., 2014). The current primary therapy for patients with advanced PCa involves chemical debulking through surgical resection or drugs that target androgen receptor signaling (Wang et al., 2018). So far, the molecular mechanism underlying occurrence, development, and metastasis of PCa remains unclear, and effective treatment methods are lacking.

Adenine nucleotide translocases (ANTs) are mitochondrial inner membrane proteins that play an integral role in the energy metabolism of tumor cells (Desquiret et al., 2006). Adenine nucleotide translocase-2 (ANT2) is expressed abundantly in mitochondria, and is closely associated with cell growth and proliferation (Battini et al., 1987; Barath et al., 1999). ANT2 is involved in maintaining the mitochondrial transmembrane potential (ΔψM); it also prevents mitochondrial membrane rupture to decrease the release of reactive oxygen species, inhibits mitochondrial apoptosis, and ultimately promotes tumor cell growth and resistance to chemotherapy (Dorner et al., 1997; Chevrollier et al., 2005). Silencing ANT2 effectively suppresses multiple types of tumors (Lu et al., 2017; Lee et al., 2019), and ANT2 phosphorylation inhibits apoptosis in PCa cells (Li et al., 2020). In addition, ANT2 knockdown promotes Apo2L/TRAIL-induced apoptosis in PCa cells through post-transcriptional upregulation of DR5 (Oishi et al., 2013). Furthermore, ANT2 can be regulated by multiple signaling pathways and is involved in regulating expression of multiple microRNAs (miRNAs) (Jang et al., 2013b, 2016; Baik et al., 2016; Hubackova et al., 2016). Although ANT2 is associated with PCa, the mechanism underlying its transcriptional regulation remains to be elucidated.

MicroRNAs not only play a regulatory role in all physiological and pathological aspects of cell biology, but also play fundamental roles in regulating gene expression at the post-transcriptional level. miRNA expression profiling and next-generation sequencing show that expression of many endogenous miRNAs is altered significantly in PCa clinical samples, and that these changes are closely related to occurrence, development, and metastasis of PCa (Catto et al., 2011; Kumar and Lupold, 2016). Notably in this regard, miR-137 acts as a tumor suppressor in a variety of cancers. In ovarian cancer cells, miR-137 inhibits tumor epithelial-mesenchymal transition (EMT) by inhibiting the AEG-1 and Snail proteins, which in turn inhibits cancer cell growth (Guo et al., 2013; Dong et al., 2016). Furthermore, miR-137 regulates multiple signal transduction pathways to inhibit pancreatic cancer (Neault et al., 2016) and is poorly expressed in clinical samples (Gu et al., 2015). Moreover, miR-137 is associated with development of PCa. Consistent with this, overexpression of miR-137-3p can inhibit cell proliferation, migration, and invasion in PCa (Zang et al., 2020). At the same time, miR-137 is involved in modulation of bicalutamide resistance in LNCaP cells (Guan et al., 2019). In addition, miR-137 inhibits glycolysis in PCa by knocking down NOX4 (Wu et al., 2019). However, the relationship between miR-137 and ANT2, as well as the effects on growth of PCa cells, remains unclear.

Here, we determine that miR-137 is involved in prostate tumorigenesis through regulation of ANT2. Our findings clarify the relationship between miR-137 and ANT2, and confirm the effect of the miR-137/ANT2 pathway during development of PCa.

## Materials and Methods

### Sample Collection and Ethics Statement

Five prostate cancer tissues and three prostatic hyperplasia samples from patients at the First Affiliated Hospital of Jinan University were enrolled. All experiments related to human samples were conducted in accordance with the Declaration of Helsinki and were approved by Jinan University. All subjects provided written informed consent prior to the start of the study. All animal procedures conformed to the Chinese National Institute of Health guidelines for human health and were approved by the Animal Research Committee of Jinan University.

### Cell Culture and Transfection

The First Affiliated Hospital of Jinan University provided DU145, LNCaP, and PC3 human PCa cells. Cells were cultured at 37°C/5% CO_2_ in RPMI 1640 medium (Gibco Laboratories, Gland Island, NY, United States) supplemented with 10% heat-inactivated fetal bovine serum (Gibco), 100 units/mL penicillin (Gibco), and 100 μg/mL streptomycin (Gibco). When cells reached 40% confluence, they were transfected with siRNAs using Lipofectamine RNAiMax (Invitrogen).

### RNA Extraction and Real-Time PCR

Total RNA was extracted using the TRIzol reagent (Invitrogen, Carlsbad, CA, United States). An EasyScript cDNA Synthesis SuperMix (TransGen Biotech, China) was used to reverse transcribe the mRNA. TransScript^®^ miRNA First-Strand cDNA Synthesis SuperMix (TransGen Biotech, China) was used for miRNA cDNA synthesis. Quantitative real-time PCR was performed using SYBR premix EX Taq II (Takara, Tokyo, Japan). Reactions were performed on a 7500 Fast Real-Time PCR System (Applied Biosystems, Foster City, CA, United States). U6 was used as a control for miRNA normalization, and GAPDH was used as a control for mRNA normalization.

### Western Blotting

Cell lysates were subjected to SDS-PAGE (Beyotime, Shanghai, China), and the resolved proteins were transferred to nitrocellulose membranes (GE Healthcare, Chicago, IL, United States). Protein bands were detected using primary antibodies specific for ANT2 and GAPDH (Abcam, Cambridge, MA, United States; 1:1000 dilution, 4°C, overnight). The membranes were then incubated for 1 h at room temperature with a secondary antibody (1:5000). The signal was visualized using enhanced chemiluminescence (ECL from Beyotime) and protein bands were imaged on a Tanon western blotting detection system (Tanon, Shanghai, China).

### Cell Counting Kit-8 Analysis

Cells were seeded in 96-well plates (three replicates) at the appropriate cell density according to the proliferation time of each cell. The plates were incubated at 37°C for 24 h. After treatment with miR-137 mimics and inhibitors (purchased from Genepharm, Shanghai, China) at different time points, 10 μL CCK-8 was added and the plate was incubated for 1–4 h. The proliferation status of the cells was evaluated by measuring absorbance at 450 nm.

### Flow Cytometry

Cells were collected by digestion with EDTA-free trypsin (Gibco), and 1–5 × 10^4^ cells were collected by washing twice with PBS (centrifugation at 2,000 rpm for 5 min). Then 2 μL Annexin V-FITC were added for 15 min at room temperature. Cells collected were carefully and mixed with 5 μL of 7-AAD and 200 μL of buffer, followed by incubation for 5–15 min at room temperature in the dark. After the incubation, the cells were passed through a 200-mesh filter and analyzed on a BD Accuri C6 flow cytometer (Accuri Cytometers Inc., Ann Arbor, MI, United States).

### Transwell Assay

Cell migration and invasion assays were performed using Transwell chambers (8.0 mm pore size; Corning, NY, United States). For the invasion assay, 100 μL of thawed Matrigel (BD Bioscience, San Jose, CA, United States) was added to each insert. After 1 h of incubation (room temperature), the liquid was aspirated. Transfected cells were starved overnight and inoculated to the upper chamber at a density of 2.5 × 10^6^ cells/mL in 200 μL medium without fetal bovine serum. After a 24 h incubation at 37°C/5% CO_2_, non-migrated/invasive cells were removed from the upper chamber using a cotton swab. Next, cells were fixed with 4% paraformaldehyde and stained with 0.5% crystal violet. Finally, the cell number was counted.

### Xenograft Mouse Model

ANT2-silenced or -overexpressing DU145 PCa cells were generated using lentiviruses. Then, the ANT2-shRNA or control cells with or without miR-137 mimics were injected subcutaneously into nude mice (*n* = 6 animals per group). After 1 month, the animals were sacrificed and tumors were collected, measured, and subjected to western blot analysis.

### Immunohistochemistry

Human tissue sections were incubated overnight at 4°C with an anti-ANT2 antibody (ab109115; Abcam, Cambridge, MA, United States) and then incubated for 1 h with a biotin-labeled anti-rabbit secondary antibody. The samples were counterstained with hematoxylin. Analysis was performed using a non-biotin polymer HRP detection system (BioGenex Laboratories, San Ramon, CA, United States).

### Luciferase Reporter Assay

The potential miR-137 binding site (wild-type or mutant) in the 3′-UTR of the human ANT2 gene was cloned into the pGL3 promoter vector (Promega, Madison, WI, United States). Luciferase activity was detected 24 h post-transfection using a dual-luciferase kit (Promega). Firefly luciferase activity was normalized against that of the luciferase control and expressed as the mean of three replicate values.

### Statistical Analyses

Data were collected from at least three independently repeated experiments and are expressed as the mean ± SD. SPSS 27.0 was used to perform statistical analyses. All data were tested for normality using the Shapiro-Wilk W method. Analyses were performed using a *t*-test (for single comparisons) or one-way analysis of variance (ANOVA; for multiple comparisons). *p* < 0.05 was considered statistically significant.

## Results

### ANT2 Is Highly Expressed in PCa Tissues

**FIGURE 1:**
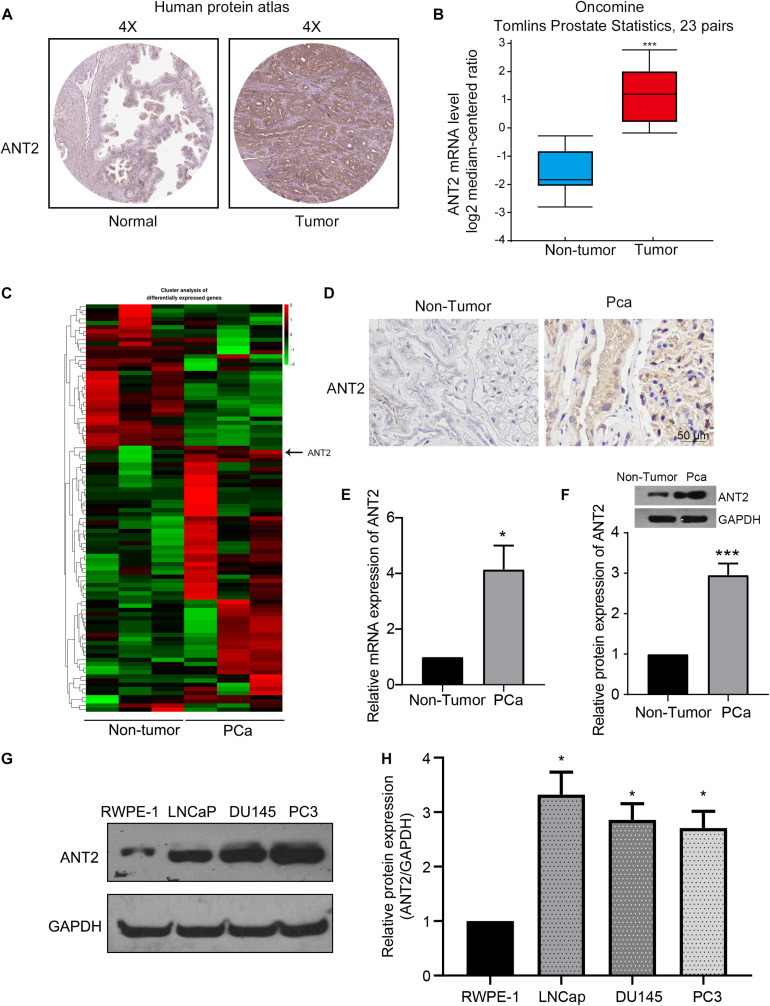
Expression of ANT2 in prostate cancer tissues. **(A,B)** By searching the Human Protein Atlas and Oncomine databases, we found that the expression of ANT2 is low in normal prostate tissues and high in prostate cancer tissues: specifically, the level of ANT2 mRNA in prostate cancers was more than 3-fold higher than in normal prostate tissues. **(C)** A heatmap comparing normal tissue with prostate cancer tissue revealed higher expression of ANT2 in prostate cancer. **(D)** Immunohistochemical staining revealed higher expression of ANT2 in prostate cancer tissues. **(E)** qPCR revealed higher expression of ANT2 in prostate cancer tissues. **(F)** Western blotting confirmed higher expression of ANT2 in prostate cancer tissues. **(G,H)** The levels of ANT2 in prostate cancer cell lines LNCaP, DU145, and PC3 were higher than those in normal prostate cells, as determined by western blot assay. *p < 0.05; ***p < 0.001.

To confirm the role of ANT2 in PCa, we first searched two online databases: the Human Protein Atlas (Ponten et al., 2008) and Oncomine (Rhodes et al., 2007). We found that ANT2 expression was three-fold higher in PCa tissue than in normal prostate tissue (Figure 1A from the Human Protein Atlas and Figure 1B from Oncomine), indicating a correlation between ANT2 expression and PCa. To further confirm this relationship, clinical samples of prostate cancer tissues and benign prostate hyperplasia samples were subjected to RNA-seq gene sequencing. A heatmap of differentially expressed genes indicated high expression of ANT2 in PCa tissues (Figure 1C). Similar results were observed after immunohistochemical staining of these samples (Figure 1D). In addition to immunohistochemical experiments, we verified expression of ANT2 in PCa by qPCR and western blotting. The results of qPCR revealed that expression of ANT2 mRNA was 4-fold higher in PCa tissues than in normal prostate tissues (Figure 1E), and western blotting revealed that expression of ANT2 protein was 2.8-fold higher in cancer tissues than in normal controls (Figure 1F). Consistent with this, western blotting confirmed that ANT2 levels were significantly higher in PCa cell lines (LNCaP, DU145, and PC3) than in normal prostate cells (Figures 1G,H). These data indicate that ANT2 is highly expressed in PCa tissues and cells.

### Inhibiting ANT2 Inhibits PCa Cell Growth and Induces Apoptosis

**FIGURE 2:**
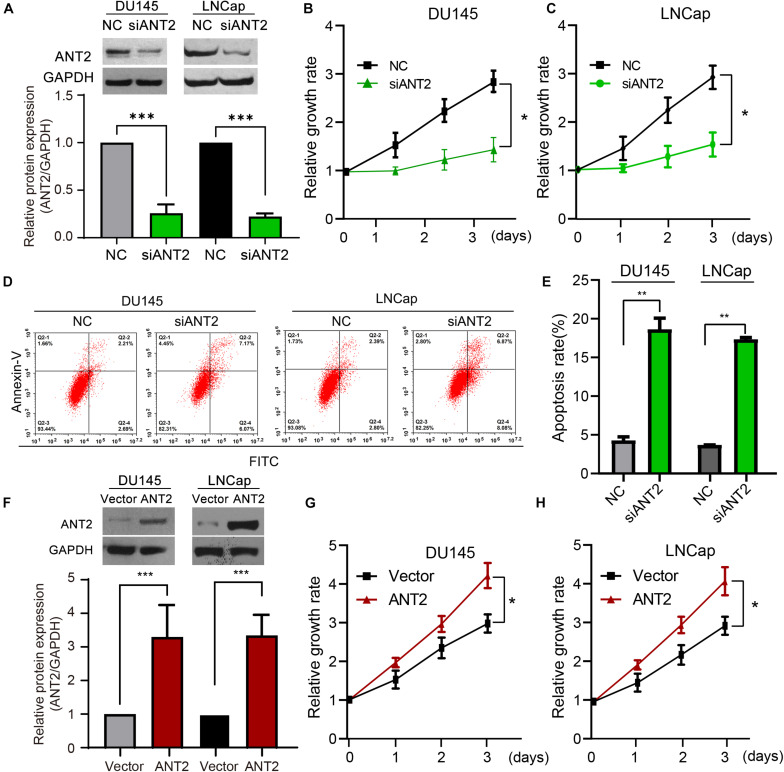
Effects of ANT2 overexpression and silencing on prostate cancer cell proliferation and apoptosis. **(A)** A siRNA was designed to target the mRNA sequence of human ANT2. The effectiveness and specificity of the siRNA (siANT2) fragment was verified by western blot assay in DU145 and LNCaP cells. **(B,C)** After ANT2 gene silencing, proliferation of DU145 and LNCaP cells decreased. **(D)** Detection of apoptosis by Annexin V–PI double-label flow cytometry: ANT2 gene silencing inhibited prostate cancer cell growth and induced apoptosis. **(E)** Apoptosis in LNCaP and DU145 cells was significantly increased after ANT2 gene silencing. **(F)** RNA was extracted from prostate cancer cells and reverse transcribed. ANT2 cDNA was amplified by PCR, cloned into the indicated vector, and sequenced. Protein expression was monitored to confirm overexpression of ANT2, and the validity and specificity of the construct were verified by western blot assay. **(G)** Overexpression of ANT2 promoted proliferation of DU145 and LNCaP cells. **(H)** Detection of apoptosis by Annexin V–PI double-label flow cytometry: overexpression of ANT2 had no significant effect on the growth or apoptosis of prostate cancer cells. *p < 0.05; **p < 0.01; ***p < 0.001; ns, no significant differences.

Next, we investigated the role of ANT2 in development of PCa. We designed and verified several siRNA fragments against ANT2 (siANT2), and selected the most effective one for the following experiments. The results revealed that knockdown of this siANT2 fragment was 80% efficient (Figure 2A). Transfection of siANT2 into DU145 and LNCaP PCa cells decreased proliferation, as measured in a CCK-8 assay (Figures 2B,C). Flow cytometry revealed that siANT2 increased the rate of apoptosis by four-fold relative to that in the NC control group (Figures 2D,E). We then constructed an ANT2 overexpression plasmid and confirmed that it increased expression of ANT2 protein in cells significantly (Figure 2F). Transfection of this plasmid promoted proliferation of PCa cells (Figures 2G,H). However, ANT2 overexpression had no effect on the rate of PCa cell apoptosis (data not shown). Taken together, these data indicate that ANT2 acts as an oncogene during development of PCa.

**FIGURE 3:**
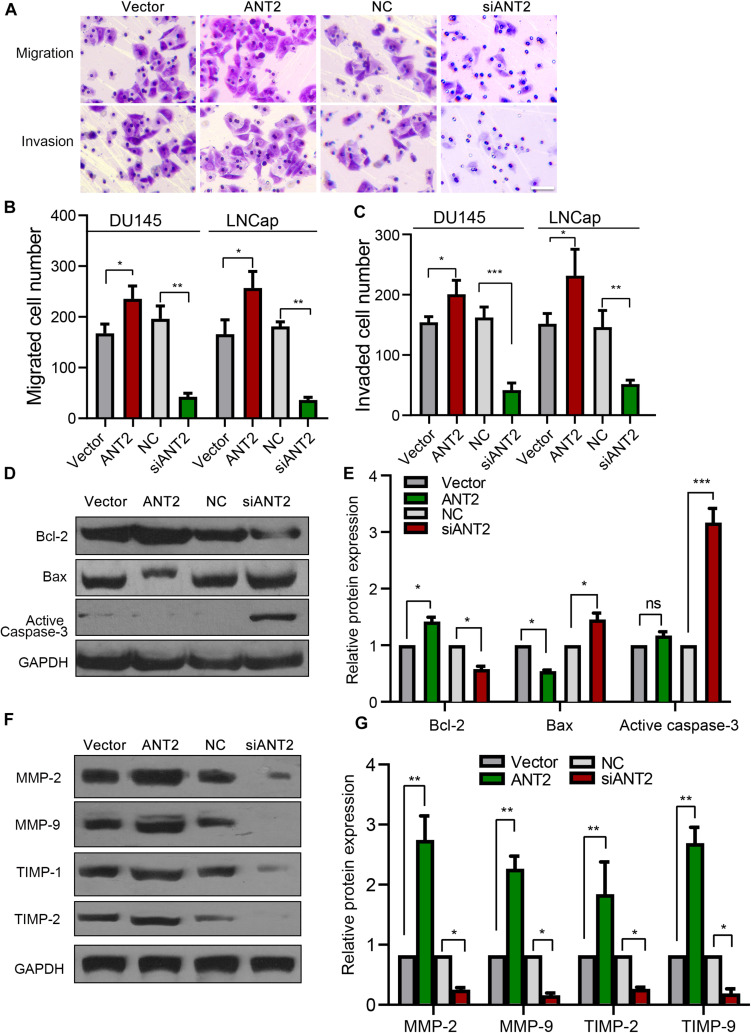
Effects of ANT2 overexpression and silencing on prostate cancer cell migration and invasion. **(A)** Cell migration and invasion were increased by overexpression of ANT2 and diminished by silencing of ANT2. Scale bar, 20 μm. **(B,C)** Overexpression of ANT2 increased migration and invasion by LNCaP and DU145 cells, and silencing of ANT2 decreased migration and invasion. **(D,E)** ANT2 overexpression caused high expression of Bax, low expression of Bcl-2, and no significant difference in Caspase-3 expression; ANT2 gene silencing caused low expression of Bax, high expression of Bcl-2, and ultra-high expression of Caspase-3. **(F,G)** ANT2 overexpression caused high expression of MMP-2, MMP-9, TIMP-1, and TIMP-2, while ANT2 silencing caused low expression of MMP-2, MMP-9, TIMP-1, and TIMP-2. *p < 0.05; **p < 0.01; ***p < 0.001; ns, no significant differences.

To investigate the influence of ANT2 on apoptotic pathways in PCa, we transfected an ANT2 plasmid or siANT2 into PCa cells and performed western blotting to detect apoptosis-related proteins such as Bcl-2, Bax, and caspase-3. ANT2 overexpression promoted expression of Bcl-2, while repressing Bax, but had no significant effect on expression of caspase-3. By contrast, siANT2 inhibited expression of Bcl-2 and promoted expression of Bax, and caspase-3 was expressed at a very high level (Figures 3D,E). Collectively, these findings indicate that silencing of ANT2 promotes apoptosis of LNCaP and DU145 cells by regulating expression of Bcl-2, Bax, and caspase-3.

### ANT2 Promotes Migration and Invasion of PCa Cells

Next, we explored the effect of ANT2 on migration and invasion of PCa cells, which we observed in a Transwell assay. Overexpression of ANT2 promoted migration and invasion, whereas silencing decreased both of these processes (Figures 3A–C). Matrix metalloproteases (MMPs) and tissue inhibitors of metalloproteases (TIMPs) play important roles in tumor metastasis and infiltration; therefore, we also examined expression of MMPs and TIMPs after transfection of PCa cells with ANT2 and siANT2. Expression of MMP-2, MMP-9, TIMP-1, and TIMP-2 was upregulated after overexpression of ANT2, but downregulated by siANT2 (Figures 3F,G). These data suggest that ANT2 regulates migration and invasion of PCa cells by modulating expression of MMPs and TIMPs.

### miR-137 Targets and Regulates ANT2

**FIGURE 4:**
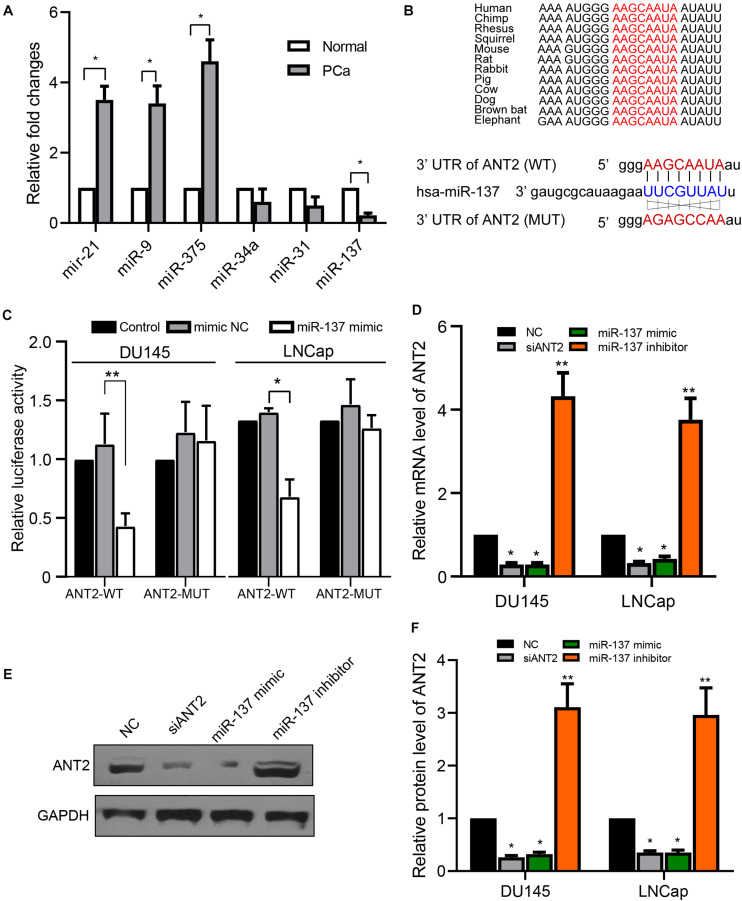
ANT2 is a target gene of miR-137. **(A)** Prostate cancer and normal tissues were subjected to qPCR to detect the indicated miRNAs. Expression of miR-137, as determined by qPCR, was reduced in prostate cancer samples. **(B)** TargetScan predicted the presence of a conserved regulatory site for miR-137 in the UTR region of ANT2. The sequences of the WT and MUT 3′-UTR of ANT2 targeted by miR-137 are shown. **(C)** In LNCaP and DU145 cells, miR-137 mimics decreased luciferase activity in ANT2 3′-UTR (wild-type) but had no statistically significant effect on luciferase activity in ANT2 3′-UTR (mutant). **(D)** DU145 and LNCaP cells were transfected with miR-137 mimic/inhibitor or siANT2 fragments. Expression of mRNA encoding ANT2 was detected by qPCR. **(E,F)** Cells were treated as in panel **(D)** and cell lysates were subjected to western blot analysis with the indicated antibodies. *p < 0.05; **p < 0.01.

Previous studies show that ANT2 can be regulated by multiple signaling pathways, as well as by multiple miRNAs (Baik et al., 2016; Lu et al., 2017). Hence, we examined the levels of different miRNAs in PCa tissues, and found that expression of miR-21, miR-9, and miR-375 was upregulated in PCa tissues relative to normal prostate tissues, whereas miR-137 was downregulated, and miR-34a and miR-31 were not affected significantly (Figure 4A). These observations suggest that many miRNAs are involved in PCa. Because miR-137 was downregulated when ANT2 was upregulated, we next sought to determine whether miR-137 has an upstream or downstream regulatory relationship with ANT2. TargetScan (Agarwal et al., 2015) predicted a conserved regulatory site for miR-137 within the 3′-UTR of ANT2, which is highly conserved among species (Figure 4B). To explore the function of this site, we constructed luciferase reporter plasmids ANT2-WT and ANT2-MUT based on these sequences (Figure 4B). As revealed by dual-luciferase reporter gene experiments in PCa cells, the miR-137 mimics inhibited the luciferase activity of ANT2-WT but not that of ANT2-MUT (Figure 4C). Furthermore, we measured endogenous expression of ANT2 mRNA after transfection of the miR-137 mimic/inhibitor, comparing the effect of siANT2 fragments. The results showed that transfection of miR-137 mimics and siANT2 fragments downregulated ANT2 mRNA significantly, while the miR-137 inhibitor upregulated ANT2 mRNA (Figure 4D). The protein level of ANT2 showed the same trend when cells overexpressed these fragments (Figures 4E,F). These data suggest that ANT2 is a direct target of miR-137.

### Antagonism of ANT2 by miR-137 Inhibits Proliferation, Migration, and Invasion of PCa Cells

**FIGURE 5:**
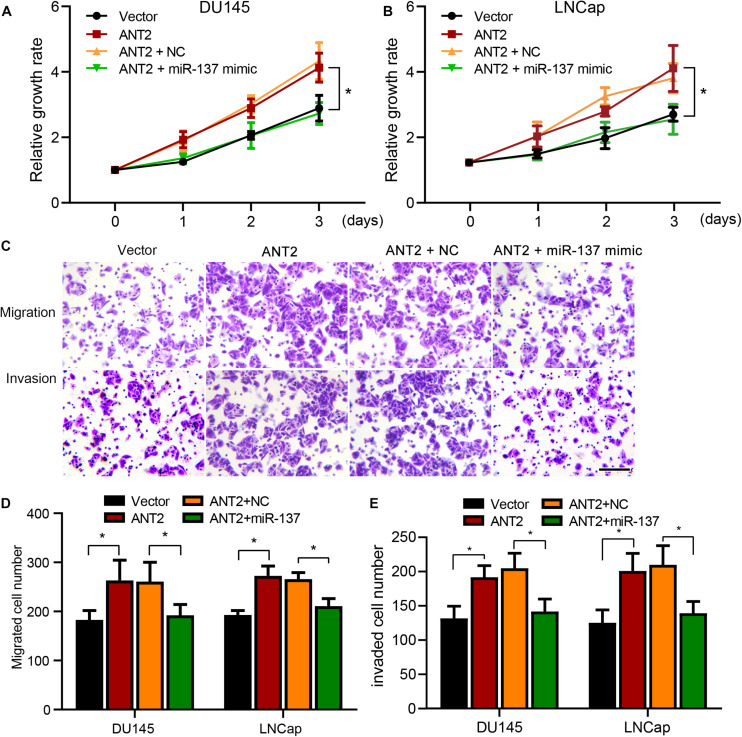
miR-137 regulates prostate cancer cells by inhibiting ANT2. **(A,B)** After treatment with miR-137 mimics + ANT2 treatment, proliferation of LNCaP and DU145 cells decreased. **(C)** Migration and invasion of LNCaP and DU145 cells were attenuated after treatment with miR-137 mimics + ANT2. Scale bar, 50 μm. **(D,E)** Migration and invasion of LNCaP and DU145 cells were increased after ANT2 overexpression but attenuated after miR-137 interference. *p < 0.05.

To explore the effect of the miR-137/ANT2 axis on cell development, PCa cells were co-transfected with the ANT2-encoding plasmid with or without the miR-137 mimics. We then assessed proliferative capacity in a CCK-8 assay. ANT2 promoted proliferation of PCa cells, an effect that was inhibited by co-expression of miR-137 mimics (Figures 5A,B). Next, we used Transwell assays to assess the metastatic and invasive ability of PCa cells. ANT2 promoted migration and invasion, an effect counteracted by co-expression of miR-137 mimics (Figures 5C–E). These findings suggest that miR-137 modulates cell proliferation, migration, and invasion by antagonizing ANT2.

### The miR-137/ANT2 Axis Regulates PCa Tumorigenesis *in vivo*

**FIGURE 6:**
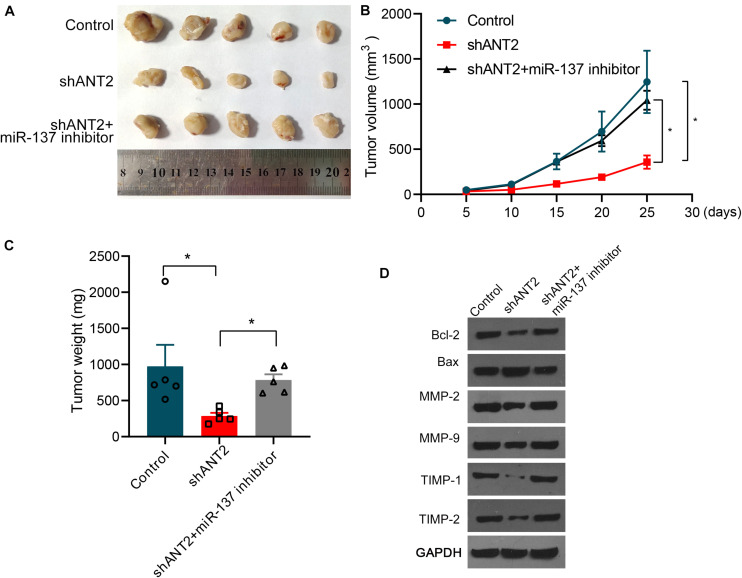
miR-137 affects prostate cancer cell growth *in vivo* via ANT2. **(A)** shANT2 DU145 cell line and control cells were injected into the lower limbs of nude mice, and tumor tissues were dissected 1 month later. DU145 was significantly tumorigenic, but the amount of tumor tissue was significantly reduced following interference with ANT2; supplementation with miR-137 inhibitors counteracted the effect of shANT2. **(B)** Tumor volume and **(C)** tumor weight in each group. **(D)** Dissected tumor tissues were subjected to western blot analysis with the indicated antibodies. *p < 0.05.

To examine the relationship between miR-137 and ANT2 *in vivo*, we performed tumorigenesis experiments in nude mice. An ANT2-deficient DU145 cell line was constructed using shANT2 lentiviruses. Next, deficient cells or control cells were injected into the lower limbs of nude mice. Mice were sacrificed and tissues were harvested 1 month later. As shown in Figure 6A, although DU145 cells were significantly tumorigenic, interference with ANT2 resulted in marked shrinkage of tumor tissue; however, tumorigenicity persisted after supplementation with the miR-137 inhibitor, which counteracted the effect of shANT2. Tumor volume and weight were significantly smaller in the shANT2 group than in the DU145 and shANT2+miR-137 inhibitor groups (Figures 6B,C). Additionally, we measured expression of Bcl-2, Bax, MMPs (MMP-2 and MMP-9), and TIMPs (TIMP-1 and TIMP-2) by western blotting. The results showed that shANT2 inhibited expression of Bcl-2, promoted expression of Bax, and inhibited expression of MMPs (MMP-2 and MMP-9) and TIMPs (TIMP-1 and TIMP-2). Co-expression of miR-137 reversed the suppressive effect of shANT2, although expression of MMPs (MMP-2 and MMP-9) and TIMPs (TIMP-1 and TIMP-2) did not differ significantly relative to that in the control group. The results of the tumorigenic assays in nude mice were consistent with those obtained from PCa cell lines (Figure 6D), suggesting that the miR-137/ANT2 signaling axis modulates *in vivo* tumorigenesis of PCa.

## Discussion

Prostate cancer is the second most prevalent malignancy in men, but no effective treatment options are available. ANT2 and miRNAs are associated with development of several cancers, but their relationship with PCa remains unclear. In this study, we confirmed that ANT2 was highly expressed in prostate tissue. Silencing of ANT2 effectively inhibited proliferation of PCa cells and induced apoptosis. Overexpression of ANT2 promoted migration and invasion by PCa cells. At the same time, we demonstrated that miR-137 targets ANT2, and that miR-137 mimics antagonize ANT2 to inhibit proliferation, migration, and invasion of PCa cells. An ANT2 inhibitor counteracted the effect of shANT2 on tumorigenesis *in vivo*. In summary, this study identifies a novel mechanism by which the miR-137/ANT2 axis regulates PCa development.

Adenine nucleotide translocases play important roles in exchange of ADP for ATP and are important transporters in the inner mitochondrial membrane (Belzacq et al., 2002). Humans possess four ANT isoforms, each of which has a distinct pattern of tissue expression. ANT2 has an anti-apoptotic profile and is overexpressed in many malignant cells (Le Bras et al., 2006; Chevrollier et al., 2011). For example, ANT2 is expressed specifically in cells that are hypo-differentiated or have the ability to re-differentiate; examples include kidney and liver cells (Liu et al., 2011; Peng and Croce, 2016). ANT2 plays a key role in the glycolytic metabolism of tumor cells (Han et al., 2016). Overexpression of ANT2 in tumor cells significantly increases the level of glycolysis, whereas overexpression of ANT3 has no effect (Han et al., 2016). Meanwhile, knockdown of ANT2 promotes induction of apoptosis by the mitochondria-targeting antitumor drug lonidamine (Le Bras et al., 2006). *In vivo*, apoptosis of human breast cancer cells can be induced by inhibiting ANT2 expression (Jang et al., 2008). Knockdown of ANT2 sensitizes breast cancer cells to Apo2L/TRAIL by upregulation of DR5 (Jang et al., 2010); this mechanism occurs in PCa cells (Oishi et al., 2013). Moreover, the combination of ANT2 knockout and clonidine induces cell apoptosis; clonidine is a mitochondria-targeted antitumor compound that has been used in clinical studies of breast, ovarian, glioma, lung, and PCa (Le Bras et al., 2006). Here, we showed that ANT2 was upregulated significantly in PCa tissues and cells, whereas knockdown of ANT2 inhibited proliferation and induced apoptosis of PCa cells (Figures 1, 2). The pro-apoptotic Bcl-2 family promotes mitochondria-mediated apoptosis, and we show that ANT2 is linked to the apoptosis of PCa cells through regulation of Bcl-2, Bax, and caspase-3, which is consistent with a previous report (Sharaf el dein et al., 2011). These findings suggest that ANT2 expression is critical for tumor cell growth, proliferation, migration, and invasion, and thus represents a potential target for tumor therapy. However, it remains unclear how ANT2 levels are regulated.

miRNAs are small non-coding RNA molecules that play multiple roles in many biological processes. They block protein expression by cleaving specific target mRNAs or by inhibiting their translation (Bartel, 2004). For example, miR-137 inhibits growth, migration, and invasion of gastric and colon cancers by targeting Cdc42 (Bartel, 2004; Peng and Croce, 2016). In addition, miR-137 targets KLF12 and MYO1c in gastric cancers to inhibit tumorigenesis (Chen et al., 2011). Meanwhile, miR-137 inhibits proliferation and migration of breast cancer cells by targeting CtBP1 and the estrogen receptor ERR (Liu et al., 2011; Du et al., 2016). These studies show that miR-137 acts as a tumor suppressor in several cancers. Meanwhile, miRNAs are associated with the staging, grading, biochemical recurrence, and metastasis of PCa (Kumar and Lupold, 2016). Recent studies show that the level of miR-137-3p is reduced in PCa, and that this miRNA restrains PCa growth, migration, and invasion by regulating the JNK3/EZH2 pathway (Zang et al., 2020). In addition, miR-137 exerts an inhibitory effect in PCa by downregulating NOX4 (Wu et al., 2019). In this study, we found that miR-137 is also a tumor suppressor in PCa, and that expression of miR-137 is diminished in prostate tumor cells. Nevertheless, the mechanism that regulates miR-137 in PCa is still not well understood. We need to confirm whether ANT2 and miR-137 have an upstream or downstream regulatory relationship. Here, we found that miR-137 targeted ANT2 directly to regulate its expression (Figure 4). In addition, targeting of ANT2 by miR-137 further regulated the proliferation, migration, and invasion of PCa cells (Figure 5). Tumorigenic assays in nude mice revealed that an miR-137 inhibitor alleviated the suppressive effect of ANT2 knockdown on tumor growth (Figure 6). However, ANT2 expression is not regulated by miR-137 alone; it can also be co-regulated by multiple miRNAs. For example, shANT2 downregulates miR-19a and miR-96 through the PI3K/Akt pathway and inhibits tumors growth in hepatocellular carcinoma cells (Baik et al., 2016). shRNA-mediated inhibition of ANT2 restores miR-636 expression, thereby downregulating Ras and inhibiting formation of hepatocellular carcinoma (Jang et al., 2013b). Inhibition of ANT2 may inhibit STAT3 activity by restoring SOCS1 expression and by downregulate miR-21, thereby exerting anticancer effects in human hepatocellular carcinoma cells (Jang et al., 2013a). Collectively, these findings show that the miRNAs targeting ANT2 are diverse, and it remains unclear whether ANT2 is regulated by multiple miRNAs in PCa.

Protease-mediated decomposition of the extracellular matrix (ECM) is an important step in cancer invasion and metastasis (Piskor et al., 2020). MMPs, members of the ECM-degrading protease family, were expressed at high levels in the PCa samples examined in this study, suggesting that activation of MMP expression in PCa promotes invasion and metastasis. Of the four membrane-type matrix metalloproteinases, MT1-MMP is most frequently overexpressed in cancer, and is frequently detected together with the activated form of MMP2. PI3K/Akt activation promotes tumor cell invasion by inducing MT1-MMP, implying that selective targeting and inhibition of the PI3K/Akt signaling pathway can attenuate the invasive potential of cancer cells significantly (Ispanovic and Haas, 2006). ANT2 interacts with pro-invasive MT1-MMP and plays a role in the coupling energy metabolism to pericellular protein hydrolysis in migrating cancer cells (Radichev et al., 2009). MMPs (MMP2 and MMP9) were also highly expressed in the PCa samples examined in this study, suggesting that expression of MMPs is activated in PCa and may enhance cancer invasion and metastasis, providing further evidence that ANT2 plays an important role in development of PCa.

## Conclusion

High expression of ANT2, a key factor involved in maintaining mitochondrial membrane potential and preventing apoptosis, enables tumor cells to survive in a hypoxic environment and improves the anti-apoptotic ability of cancer cells. ANT2 plays very important roles in growth, migration, and invasion of PCa cancer cells. miR-137 is associated with PCa, although its relationship with ANT2 needs to be examined in depth. In this study, we are trying to depict the following scenario: miR-137 expression is suppressed during PCa development, leading to upregulation of its target protein ANT2 and thereby upregulating glycolysis in tumor cells with adequate energy supply, ultimately promoting their growth. Elevation of miR-137 levels could inhibit expression of ANT2, downregulate glycolysis to cut off mitochondrial energy transport and, ultimately, suppress tumor growth. Collectively, our findings show that ANT2 is an important new target for miR-137-mediated regulation of PCa, providing a new theoretical and experimental basis for elucidating the pathogenesis of PCa and designing targeted therapies.

## Data Availability Statement

The raw data supporting the conclusions of this article will be made available by the authors, without undue reservation.

## Ethics Statement

The studies involving human participants were reviewed and approved by the Jinan University. The patients/participants provided their written informed consent to participate in this study. The animal study was reviewed and approved by Animal Research Committee of Jinan University.

## Author Contributions

YL, WZ, and BP conceived the project. HZ, NC, and ZD conducted the experiments. YM, LD, and GC helped with biochemistry experiments and data analysis. BP and WZ wrote the manuscript. All authors contributed to the article and approved the submitted version.

## Conflict of Interest

The authors declare that the research was conducted in the absence of any commercial or financial relationships that could be construed as a potential conflict of interest.

## Publisher’s Note

All claims expressed in this article are solely those of the authors and do not necessarily represent those of their affiliated organizations, or those of the publisher, the editors and the reviewers. Any product that may be evaluated in this article, or claim that may be made by its manufacturer, is not guaranteed or endorsed by the publisher.
